# Vertebroplasty and vertebroplasty in combination with intermediate bilateral pedicle screw fixation for OF4 in osteoporotic vertebral compression fractures: a retrospective single-Centre cohort study

**DOI:** 10.1186/s12893-019-0646-x

**Published:** 2019-11-27

**Authors:** Weiyang Zhong, Xinjie Liang, Xiaoji Luo, Zhengxue Quan

**Affiliations:** 1 Department of Orthopaedic Surgery, The First Affiliated Hospital of Chongqing Medical University, Chongqing, China; 2 Department of Pain Management, The First Affiliated Hospital of Chongqing Medical University, Chongqing, China

**Keywords:** Vertebral osteoporotic compression fractures, Thoracolumbar spine, Vertebroplasty, Short segmental instrumentation

## Abstract

**Background:**

Although various studies have described the outcomes and complications of each treatment for OF 4 in osteoporotic vertebral compression fractures (OVCFs), there is still no consensus on the optimal treatment regimen. This study aimed to investigate the clinical effect of OF 4 in patients with OVCFs treated with percutaneous vertebroplasty (PV) compared with PV in combination with intermediate bilateral pedicle screw fixation (IBPSF).

**Methods:**

A total of 110 patients with OF 4 in OVCFs from January 2011 to December 2013 were reviewed retrospectively and divided into two groups (group A: PV, group B: PV + IBPSF). According to the guidelines of the German Society for Orthopaedics and Trauma (DGOU), OF 4 consists of 3 fracture types. The clinical and radiographic assessments were observed preoperatively, postoperatively, and during follow-up.

**Results:**

The patients were followed for an average of 60.50 ± 15.20 months (group A) and 58.20 ± 17.60 months (group B) without significant differences. No significant differences were found in BMD, BMI and cement volume between the two groups, but differences were found for operation time, blood loss, and hospitalization time. The VAS and ODI scores improved better significantly at the final follow-up in group B but not in group A. Compared with the preoperative values, the postoperative kyphosis angle and loss of fractured segment height significantly improved, but the difference between the groups was significant after 3 months postoperatively. The loss of angular correction and fractured segment height in group A were greater than those in group B. A total of 15 cases of cement leakage were observed in group A and 8 cases in group B, and no complications or revision surgeries were observed in either group. Thirteen new fractures occurred (11 in group A and 2 in group B), which was significant.

**Conclusion:**

PV with IBPSF could provide effective restoration and maintenance of fractured segment height and segment alignment as well as a lower rate of complications of OF 4 in OVCFs.

## Background

Osteoporotic vertebral compression fractures (OVCFs), represent a common fracture event, and morbidity and mortality rates gradually increase in the elderly population. An estimated 1.4 million new fractures occur every year worldwide [1, 2]. OVCFs have been treated with conservative management, such as bed rest, analgesics, and braces, while the surgical treatments include PV, percutaneous kyphoplasty (PK) or segmental instrumentation. According to the guidelines of the DGOU, OF 4 is described as the loss of integrity of the vertebral frame structure, vertebral body collapse, or pincer-type fracture. This subgroup consists of 3 fracture types, they represent unstable fractures, and intravertebral vacuum clefts are often visible. The following surgical management is recommended for OF 4: posterior instrumentation with cement augmentation, long-segment posterior instrumentation, or posterior instrumentation with additional anterior reconstruction [3, 4]. These treatments are associated with management and social problems, and there is still no consensus for the best treatment recommendation for OF 4 in OVCFs. Furthermore, in OVCFs in elderly individuals who have medical comorbidities, the incidence of complications during surgery is extremely high, and there is even an increasing rate of disability and mortality [5, 6]. Hence, individualized therapeutic options should be considered and recommended.

In our study, according to the guidelines of the DGOU, OF 4 represent unstable fractures. The purpose of this research was to investigate the clinical effect of PV compared with PV + IBPSF for the treatment of OF 4 in OVCFS patients.

## Methods

### Patient selection

**Fig. 1:**
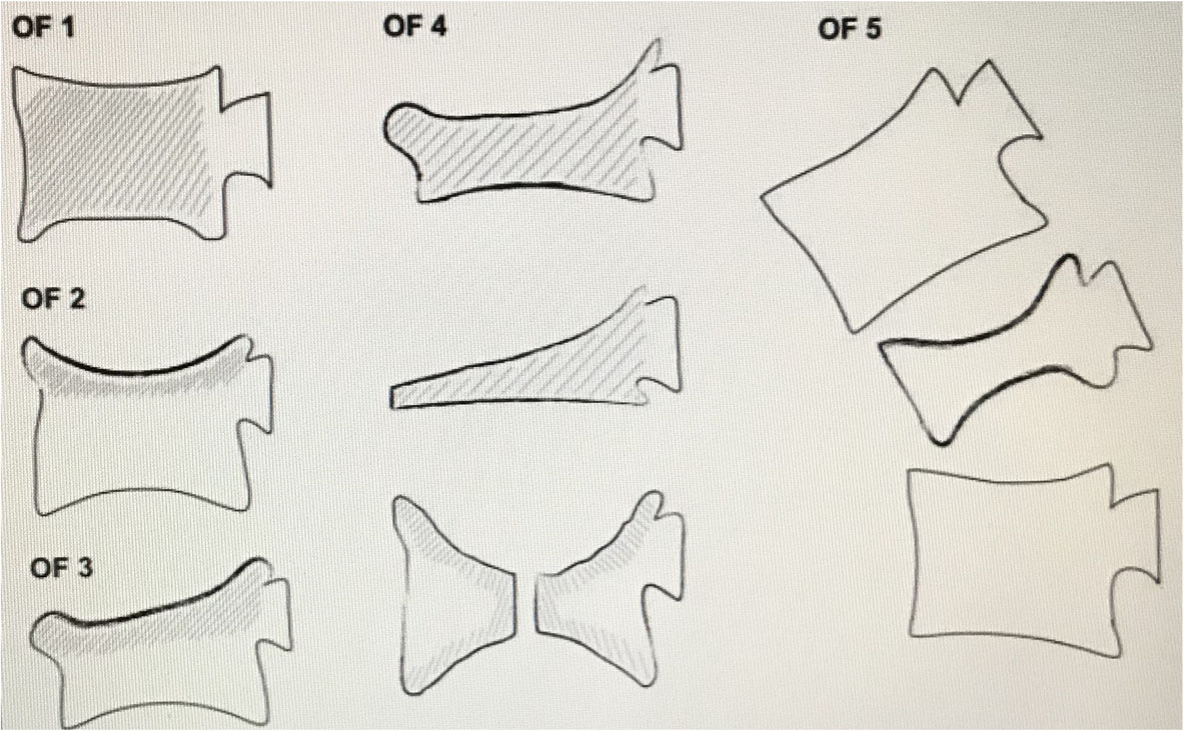
Presentation of the 5 types of OFs

From January 2011 to December 2013, a total of 110 patients with OVCFs were treated and reviewed retrospectively in our department. Two treatment plans were involved in the routine clinical care. When communicating with patients before surgery, the advantages and disadvantages of the two types of management were fully introduced, so the patients could choose the right treatment for themselves. All surgical procedures were performed by the same senior surgeon. In group A, 70 patients received PV, and in group B, 40 patients underwent PV in combination with IBPSF. The inclusion criteria were as follows: acute OVCFs classified as OF 4 (Fig. 1) [1, 2]; fractures with osteoporosis (T score < − 2.5), fractures confirmed by magnetic resonance imaging (MRI); and a minimum follow-up of 2 years. The exclusion criteria were as follows: metastatic fractures, primary tumours or high-energy trauma fractures.

### Surgical methods

In group A: The patient was placed in a prone position. PV was performed bilaterally or unilaterally under C-arm X-ray guidance (Ziehm Imaging Systems, Ziehm Imaging GmbH) using local anaesthesia. A bone needle was percutaneously inserted into the posterior one-third of the fractured vertebral body. The working cannula was transpedicularly advanced into the vertebral body. Afterwards, polymethylmethacrylate (PMMA) was slowly injected into the fractured body. The amount of surgical haemorrhage, surgical time and cement volume were recorded accordingly.

In group B: The patient was placed in a prone position after the administration of conventional tracheal anaesthesia. After a posterior midline incision was made, PV was performed on the fractured vertebral body, and pedicle screws were fixed (called intermediate bilateral pedicle screw fixation, IBPSF). The device was properly locked, and C-arm X-rays were used to ensure the accuracy of the screw fixation. After surgery, negative pressure drainage was implemented, and the incision was closed.

### Postoperative management

The drainage tube was removed when drainage was less than 50 ml/day, and the patients in group B were recommended to wear a brace for 6 to 8 weeks. All patients were treated strictly with anti-osteoporosis drugs preoperatively and postoperatively and were evaluated clinically and radiologically at 1 week and at 3, 6 and 12 months after surgery and once a year thereafter.

### Outcome indexes

For all patients, the following data were observed preoperatively, postoperatively, and during follow-up: (1) the operation time, amount of surgical haemorrhage, hospitalization time, BMD, body mass index (BMI), amount of cement leakage, and presence of new fractures; (2) VAS and ODI scores; (3) fracture segment height was measured at the point of maximal collapse of the fractured vertebral body, and height loss (%) was calculated with the following formula: {[(lower vertebral height + upper vertebral height)/ 2 − affected vertebral height]/[(lower vertebral height + upper vertebral height)/2]} × 100. Vertebral height was measured at the point of maximal collapse of the fractured vertebral body; (4) the local and thoracolumbar kyphosis angle: local kyphotic angle (°) was measured using Cobb’s method between adjacent vertebrae of the fractured vertebra.

### Statistical analysis

All statistical data were analysed with SPSS version 22.0 statistical software (SPSS, Inc., Chicago, IL, USA). Quantitative data are presented as the means and standard deviations. Repeated measures ANOVA was used for the statistical analysis of differences in mean values, and the chi-squared test was used for categorical data. Statistical significance was defined as a *p*-value < 0.05.

## Results

### Clinical features of the patients

The patients were followed for an average of 60.50 ± 15.20 months in group A and 58.20 ± 17.60 months in group B, without a significant difference (*P* > 0.05). All the patients were retired in two groups. No significant differences were found in BMD, BMI, smoking status, diabetes mellitus (DM), hypertension, coronary disease or cement volume between the two groups, but significant differences were found for the operation time, blood loss, and hospitalization time. VAS and ODI scores improved significantly at the final follow-up in group B compared with the scores in group A (Table 1).

**Table 1:** Clinical data and clinical outcomes of the two groups

	Group A	Group B	*P* value
No. of patients (n)	70	40	
Male/female (n)	21/49	16/24	
Mean age (years)	76.13 ± 15.20	75.08 ± 17.60	0.612
Follow-up (months)	60.50 ± 15.20	58.20 ± 17.60	0.650
BMD	−3.9 ± 0.8	−4.0 ± 0.6	0.521
BMI	23.2 ± 4.2	24.8 ± 3.3	0.801
Hospitalization time (days)	3.2 ± 1.5	6.0 ± 2.5	0.0001
Surgery time (minutes)	30.50 ± 10.50	70.70 ± 20.60	0.0001
Smoker/non-smoker	9/61	5/35	0.411
With/without DM	15/55	8/32	0.320
With/without hypertension	35/35	18/22	0.895
With/without coronary disease	11/59	7/33	0.305
Surgical haemorrhage (ml)	10.80 ± 5.20	100.30 ± 30.50	0.0001
Cement volume (ml)	5.09 ± 0.65	5.15 ± 0.35	0.765
VAS score			
before treatment	6.5 ± 1.88	7.0 ± 1.35	0.554
final follow-up	2.60 ± 0.55*	1.01 ± 0.25*	0.012
ODI score			
before treatment	41.15 ± 5.02	40.98 ± 3.53	0.574
final follow-up	40.75 ± 3.52	4.83 ± 1.15*	0.0001

*before treatment vs final follow-up, *P* < 0.05

### Radiographic outcomes

**Fig. 2:**
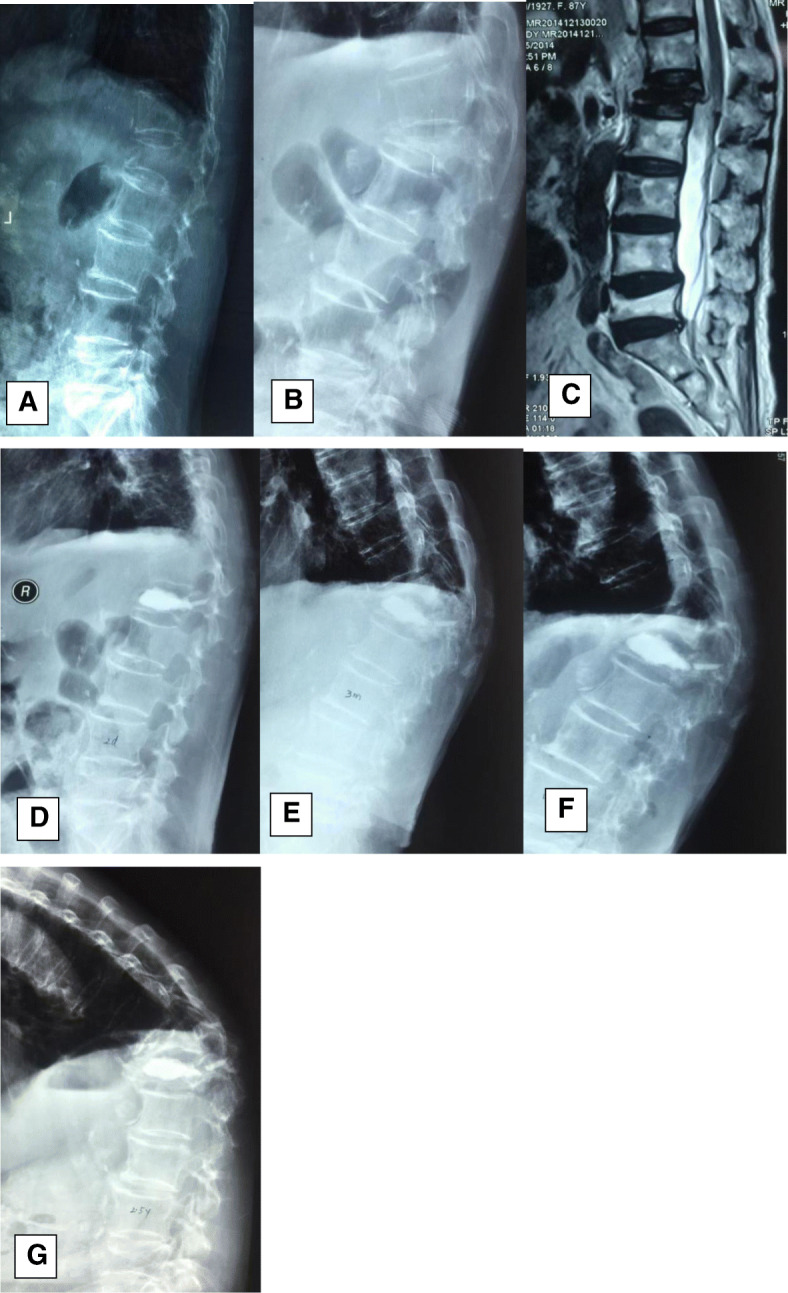
An 87-year-old female patient with an L1 osteoporotic fracture; conservative treatment failed, and then she was treated surgically with PV. One month after the operation, an adjacent-segment fracture occurred. **a** On the injured day, the patient accepted conservative treatment. **bc** The first admission X-ray and MRI showed a lumbar compression fracture at L1, and she underwent PV treatment. **defg** At the follow-up times of 3 months, 1 year, and 2.5 years postoperatively, X-ray showed vertebral collapse at L1, and a new adjacent-segment fracture occurred at T12; the kyphosis deformity deteriorated. However, the patient refused surgery, and she suffered from chronic back pain

**Fig. 3:**
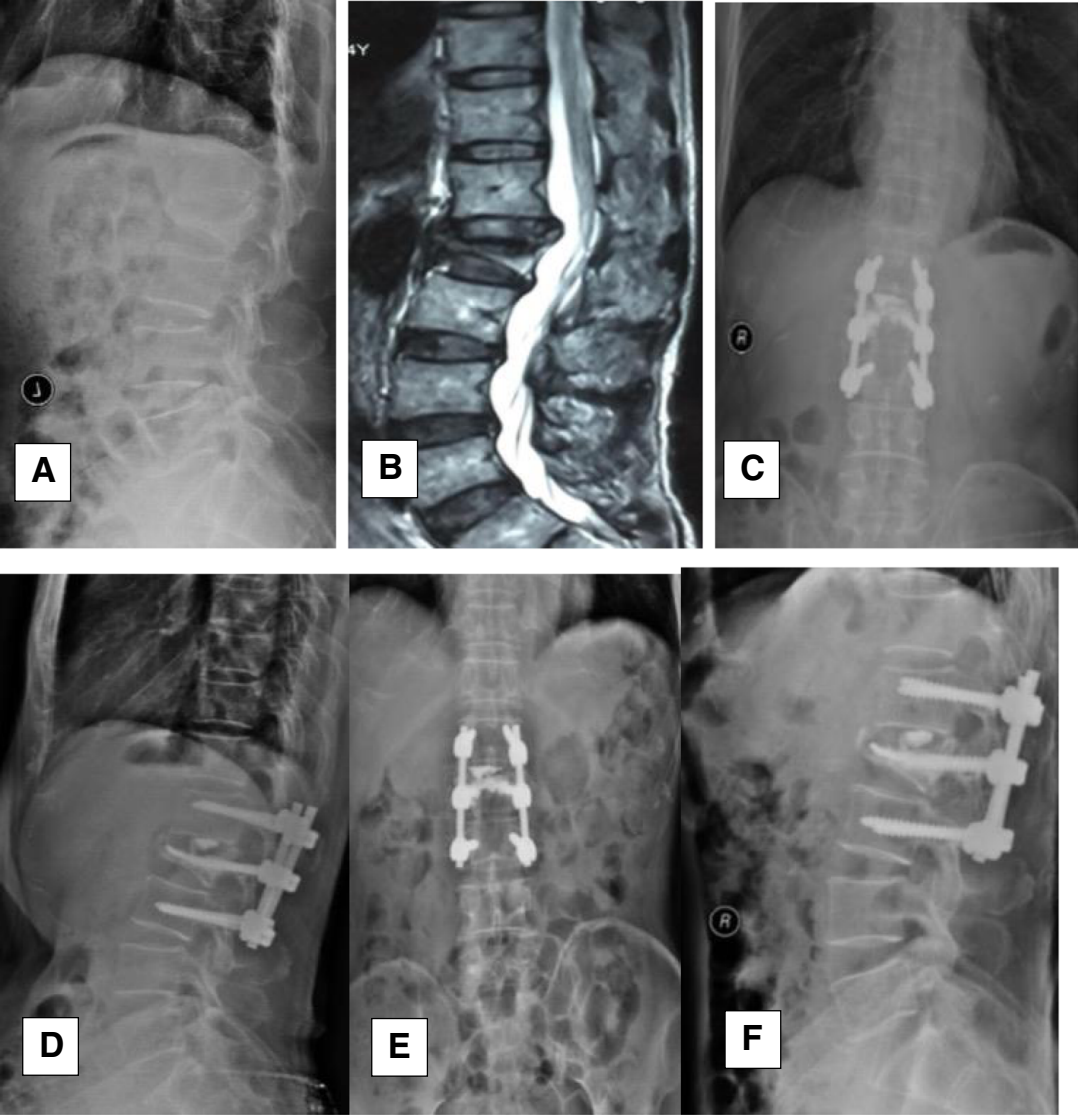
A 70-year-old male patient with an L2 osteoporotic fracture was treated surgically with PV combined with IBPSF. **ab** The first admission X-ray and MRI showed a lumbar compression fracture at L2, and he underwent surgical treatment. **cdef** At the 1-month follow-up and final follow-up, X-ray showed that the fractured vertebral height had been restored, kyphosis was sufficiently corrected, and low levels of cement leakage were observed

Compared with the preoperative values, the postoperative kyphosis angle and loss of fracture segment height significantly improved (Table 2), but the difference between the groups was significant (*P* < 0.05) after 3 months postoperatively. The loss of angular correction and fractured segment height in group A was greater than that in group B (*P* < 0.05) (Figs. 2 and 3).

**Table 2:** The radiographic outcomes

	Group A	Group B	P value
Local kyphosis angle (°)			
before treatment	25.77 ± 5.49	26.97 ± 6.80	0.301
immediately post-op	5.20 ± 2.05*	4.05 ± 1.50*	0.020
3 months post-op	10.31 ± 3.04*	5.25 ± 2.35*	0.0001
final follow-up	15.31 ± 5.50*	7.25 ± 4.85*	0.0001
Loss of fracture vertebral body height			
before treatment	30.80 ± 11.70	31.3 ± 10.90	0.685
immediately post-op	15.09 ± 5.50*	9.20 ± 3.25*	0.0001
3 months post-op	18.70 ± 6.45*	9.35 ± 4.05	0.0001
final follow-up	22.51 ± 7.85*	10.03 ± 2.40	0.0001
Cement leakage	15 (21.43%)	8 (20%)	0.750
New fractures	11 (15.7%)	2 (5%)	0.0001

*before treatment vs follow-up, *P* < 0.05

### Complications

Two cases of superficial wound infection using antibiotics and debridement were recorded in group B. Cement leakage was observed in 15 patients in group A and 8 patients in group B, and no complications or revision surgeries were observed in either group. New fractures occurred in 13 patients (11 patients in group A and 2 patients in group B), and the difference between groups was significant (*P* < 0.05) (Table 2).

## Discussion

Although OVCFs are considered stable injuries and patients are treated conservatively with bed rest, analgesics, braces, early rehabilitation and osteoporosis treatment, these fractures tend to progressively collapse, resulting in chronic pain, progressive kyphosis, or even delayed paralysis. Surgical management is preferred for vertebral height restoration, kyphosis correction and pain relief [1, 4, 7, 8]. PV or PK are widely performed because of their advantages. However, new OVCFs are common in patients who have undergone PV, thus reducing patient satisfaction. The incidence of new OVCFs in group A was 15.7%, while the incidence of new OVCFs (both adjacent and non-adjacent) was 5.5–52.0% in group B [8–14]. Although PV acquired good results in the short term, the VAS score, segment height and kyphosis angle in the patients treated with PV combined with IBPSF were significantly improved after surgery. Furthermore, the loss of segment height and kyphosis angle in group A were greater than those in group B, and they are strong risk factors for new OVCFs; however, VAS scores were decreased, thus disappointing the patients [15–19].

In our study, elderly patients did not strictly adhere to the medical advice after PV treatment. They typically began life activities without using anti-osteoporosis drugs or wearing a brace when needed, which could be reasons why the vertebral height collapsed quickly and the kyphosis deformity deteriorated, resulting in PV failure. In addition, because the patients’ children were busy with work and worried about the cost of time, about money and about the surgical risks, the patients with many comorbidities preferred simple surgical management to achieve rapid and good effects and to avoid burdens, which was why these patients did not undergo PK or PV reinforcement with IBPSF [15, 17].

Our study suggested that PV reinforcement with IBPSF could help provide better kyphosis correction, which more effectively restores fractured segment height, thus improving the biomechanical stability of unstable OVCFs [10–14, 20]. Traditional short-segment 4 pedicle screw fixation is widely used. However, many studies have demonstrated that intermediate bilateral pedicle screw fixation provides a stiffer construct and less von Mises stress on the pedicle screws and rods compared with traditional fixation. Furthermore, this procedure increases the stability of short-segment fixation and offers biomechanical support by using intermediate screws for the fractured vertebrae. In most OVCFs, although the vertebral body collapses severely, the pedicles are relatively intact and could stabilize the fractured vertebra. In our study, there were no complications, such as screw loosening or breakage, or fractured segment height loss or kyphosis correction loss. This result indicated that PV with IBPSF significantly improved the fractured segment height and provided better correction of spine alignment. However, the screw instrumentation of the fractured vertebrae was more difficult and risky than usual and required strict professional surgeon training and imaging technique support. Furthermore, the screws are still more risky to be loosened in the osteoporotic bone while the long constructions of revision suregry are needed if the surgery failed. Hence, the strict anti-osteoporosis treatment and the strict follow-up are very important.

## Conclusions

PV with IBPSF could provide effective restoration and maintenance of fracture segment height and segment alignment and a lower rate of OF 4 complications in OVCFs. However, we want to declare that there existed several limitations in this study. First, the study didn’t consider the distribution of three types in each group which was associated with bias. Second, the patients were performed by two different surgeries and the postoperative management were not exactly the same which was also associated with bias. Third, the retrospective nature of the small-sample study may be associated with bias. In the future, the prospective, randomized studies with long-term follow-up periods are needed.

## Data Availability

The datasets generated and/or analysed during the current study are not publicly available due to the data is confidential patient data but are available from the corresponding author on reasonable request.

## Abbreviations

BMD: Bone mineral density
BMI: Body mass index
DGOU: The German Society for Orthopaedics and Trauma
IBPSF: Intermediate bilateral pedicle screw fixation
MRI: Magnetic resonance imaging
ODI: Oswestry Disability Index
OVCFs: Osteoporotic vertebral compression fractures
PK: Percutaneous kyphoplasty
PMMA: Polymethylmethacrylate
PV: Percutaneous vertebroplasty
VAS: Visual analogue scale
